# Comparison of Visual Attention to Anatomical Images Between New Zealand European and Pasifika Anatomy Students

**DOI:** 10.1007/s40670-025-02425-5

**Published:** 2025-05-28

**Authors:** Ilaisaane Foli Fakapulia, Yusuf Özgür Çakmak, Latika Samalia, Erik Wibowo

**Affiliations:** 1 Department of Anatomy, University of Otago, Dunedin, 9016 New Zealand; 2 School of Medical Sciences, Faculty of Medicine and Health, The University of Sydney, Sydney, 2006 Australia

**Keywords:** Visual attention, Pasifika students, New Zealand European students, Tapu, Cultural factors, Anatomy

## Abstract

Anatomy education includes topics that are sensitive to some students due to their ethno-cultural background. Using a Tobii eye-tracker, we assessed the visual attention of New Zealand European (NZE) and Pasifika anatomy students to anatomical images. Pasifika students fixated less on surface anatomy images of the hand, male perineum, and gluteal regions than NZE students. Pasifika students fixated more on plastic model images of the gluteal, foot, and male perineum, as well as on images of the shoulder of human donors than NZE students. Ethnic background may influence the anatomy learning experience.

## Introduction

Many universities have students with diverse ethnic backgrounds. Our department has 6–19% Pasifika and 25–51% New Zealand European (NZE) students in undergraduate anatomy courses every year [1]. “Pasifika” people in New Zealand are people who migrated to, or descendants of people who moved to New Zealand from the Pacific Islands [2]. In learning anatomy, NZE and Pasifika students may differ in their in-class engagement because there are topics that are considered *tapu* (sacred/forbidden) in Pacific cultures, such as nudity in surface anatomy, reproductive organs, head region, and deceased human materials [3, 4]. Obviously, eliminating all *tapu* content from anatomy courses is not possible. Potentially, Pasifika students who are uncomfortable with these may avoid looking at images that they consider *tapu*. In doing so, they may be disengaged when learning those topics. However, currently there is no objective data showing ethnic differences in visual attention to anatomical images.

Here, we aim to compare the perceptions on anatomy learning and visual attention to anatomical images between NZE and Pasifika anatomy students, using an eye-tracking device. The eye-tracking device is useful for educational research as it measures visual attention with high temporal and spatial sensitivities [5]. For example, using an eye-tracker, Zumwalt et al. [6] show that anatomy students fixate longer on familiar or cognitively salient anatomy images. Various factors can influence visual attention, such as the nature of stimuli [5]. We expect the findings from this study can be used as evidence for the need to improve anatomy teaching delivery to a diverse cohort of students.

## Activity

This research was approved by the University of Otago’s Human Ethics Committee (D23/158). In 2023, our department had 74 Pasifika and 346 NZE students. In our previous online study, we recruited ~ 50% of Pasifika students in our department [7]. Since this research involved in-person tasks, the recruitment rate was expected to be lower. Thus, we aimed to recruit approximately 40% of Pasifika students and an equal sample size of NZE students. This sample size should allow us to assume data normality [8].

Using a convenience sampling method, we sent an email invitation to Pasifika and NZE students in our department, and we recruited 31 NZE and 31 Pasifika students. Pasifika students were the ones who identified as at least one of the following ethnicities: “Samoan,” “Cook Islands Māori,” “Tongan,” “Niuean,” “Tokelauan,” “Fijian,” and “Other Pacific Peoples.”

### Eye-Tracking Experiment

Each participant viewed a slideshow in front of a computer while wearing an eye-tracking device (Tobii eye-trackers wireless glass II, Sweden). Each slide lasted 15 s and displayed anatomical images (deceased human donors, plastic model, and surface anatomy) of a body region horizontally. All surface anatomy models were of European ethnicity. The orders of the body regions (17 in total) and the horizontal placement of image types were randomized. Before viewing the slideshow, participants were uninformed that their visual attention was being tracked. After the slideshow, they were debriefed and given a chance to withdraw from the study.

Three eye-tracking measures were analyzed: (1) total number of fixations in 15 s, (2) first–second fixations (FSF, the number of fixations within 1 s after being exposed to a slide, (3) latency (the time to fixate on an image after being exposed to a slide).

### Questionnaires

After viewing the slideshow, participants filled a standard demographic questionnaire, and questions related to their perception of anatomy learning. The questions, rating scales, and body regions assessed are listed on Table 1. Each participant received a $50 NZD gift voucher at the end of the study.

**Table 1:** Self-reported data of participants on their perception on anatomy learning. Data are presented as mean (standard deviation). Each question is rated from 0 to 10, with 10 being the most positive response (e.g., very appropriate, very comfortable) and 0 being “not at all”

	NZE	Pasifika	Test statistics	*P* valu**e**
How appropriate to your cultures are the following anatomy topics taught in lectures?				
Head and neck	7.81 (3.06)	8.45 (1.65)	*t*(60) = − 1.03	0.30
Upper and lower limbs	8.10 (3.05)	8.26 (1.51)	*t*(60) = − 0.26	0.79
Thorax	8.16 (2.93)	8.58 (1.48)	*t*(60) = − 0.71	0.48
Abdomen	8.00 (2.96)	7.94 (1.86)	*t*(60) = 0.10	0.91
Pelvis	7.65 (2.93)	6.45 (3.13)	*t*(60) = 1.55	0.12
Perineum	7.81 (3.09)	7.35 (2.72)	*t*(60) = 0.61	0.54
Gluteal region	7.74 (3.09)	6.90 (2.86)	*t*(60) = 1.11	0.27
How comfortable are you in openly discussing about the following anatomy topics in class?				
Head and neck	9.80 (0.66)	9.32 (1.14)	*t*(59) = 1.99	0.051
Upper and lower limbs	9.77 (0.67)	9.48 (0.85)	*t*(60) = 1.49	0.14
Thorax	9.74 (0.73)	9.23 (1.59)	*t*(60) = 1.64	0.11
Abdomen	9.42 (1.90)	8.84 (1.80)	*t*(60) = 1.24	0.22
Pelvis	8.97 (1.40)	7.90 (2.27)	*t*(60) = 2.22	0.03
Perineum	9.29 (1.53)	8.23 (2.43)	*t*(60) = 2.06	0.04
Gluteal region	9.32 (1.35)	8.52 (2.00)	*t*(60) = 1.86	0.07
How comfortable are you in viewing and studying the following anatomical regions using real human body parts (e.g., cadavers/prosections/plastinated models)?				
Head and neck	8.39 (1.87)	8.16 (1.93)	*t*(60) = 0.47	0.64
Upper and lower limbs	9.03 (1.22)	9.00 (1.29)	*t*(60) = 0.10	0.92
Thorax	8.90 (1.38)	9.06 (1.34)	*t*(60) = − 0.47	0.64
Abdomen	8.48 (2.10)	8.68 (1.49)	*t*(60) = − 0.42	0.68
Pelvis	7.81 (2.01)	7.58 (2.38)	*t*(60) = 0.40	0.69
Perineum	8.32 (1.80)	8.13 (2.09)	*t*(60) = 0.39	0.70
Gluteal region	8.35 (1.84)	8.06 (2.00)	*t*(60) = 0.59	0.55
How comfortable are you in viewing and studying the following anatomical regions using plastic models?				
Head and neck	9.48 (1.26)	9.29 (1.13)	*t*(60) = 0.63	0.53
Upper and lower limbs	9.61 (0.92)	9.35 (1.02)	*t*(60) = 1.04	0.30
Thorax	9.52 (0.96)	9.26 (1.24)	*t*(60) = 0.92	0.36
Abdomen	9.48 (1.00)	9.26 (1.15)	*t*(60) = 0.83	0.41
Pelvis	9.26 (1.26)	8.90 (1.47)	*t*(60) = 1.02	0.31
Perineum	9.52 (1.00)	9.00 (1.34)	*t*(60) = 1.72	0.09
Gluteal region	9.48 (1.09)	9.00 (1.37)	*t*(60) = 1.54	0.13
How comfortable are you in viewing and studying the following anatomical regions using reading materials (e.g., textbooks or lecture slides)?				
Head and neck	9.77 (0.81)	9.32 (1.25)	*t*(60) = 1.69	0.10
Upper and lower limbs	9.84 (0.52)	9.42 (1.09)	*t*(60) = 1.93	0.06
Thorax	9.81 (0.54)	9.42 (1.09)	*t*(60) = 1.77	0.08
Abdomen	9.81 (0.54)	9.48 (1.00)	*t*(60) = 1.58	0.12
Pelvis	9.45 (1.00)	9.26 (1.15)	*t*(60) = 0.71	0.48
Perineum	9.68 (0.70)	9.35 (1.11)	*t*(60) = 1.37	0.18
Gluteal region	9.68 (0.79)	9.42 (1.03)	*t*(60) = 1.10	0.27
To what extent do cultural factors (e.g., tapu/taboos) impact the way you view cadavers as learning tools?	2.55 (2.53)	7.03 (2.23)	*t*(60) = − 7.41	< 0.001

### Data Analyses

Data analyses were conducted using the statistical software package (SPSS version 29, IBM). An independent *t*-test was used to compare questionnaire data based on ethnicities. Two-way ANOVA was used to determine the main effects and interaction effects of ethnicity and image type on visual attention variables. Biological sex was not included in our analyses because preliminary analyses showed that biological sex was not associated with the number of fixations on any image. If the interaction effect was significant, a Tukey post hoc test followed. *P* < 0.05 was considered significant.

## Results and Discussion

### Demographics

On average, NZE students were 20.3 ± 0.7 years old, while Pasifika students were 22.1 ± 2.4 years old. Additionally, 23 (74.2%) NZE and 17 (54.8%) Pasifika students were female. The top three Pacific ethnicities were Samoan (22.6%), Tongan (25.8%), and Fijian (29%).

### Perception of Anatomy Learning

Pasifika students reported lower comfort in openly discussing pelvis (*P* = 0.030) and perineum (*P* = 0.044) anatomy in class than NZE students (Table 1). This is somewhat expected because young Pacific peoples express apprehension when discussing sexual topics with other people [9].

Pasifika students had a stronger perception that cultural factors impacted the way they viewed deceased donors as learning tools than NZE students (*P* < 0.001). This is consistent with another study indicating how Pasifika students may feel conflicted when they need to perform human dissection as they view elderly people with reverence and filial piety [10].

Our finding also relates to how the dead are respected in Pacific cultures. A widely held belief across many Pacific cultures is that the deceased should be buried whole and intact, including all their organs [4]. This stems from the notion that the dead are still alive even in the afterlife. Therefore, organs that are removed must be handled with great respect and buried appropriately [4].

### Visual Attention to Anatomical Images

**Fig. 1:**
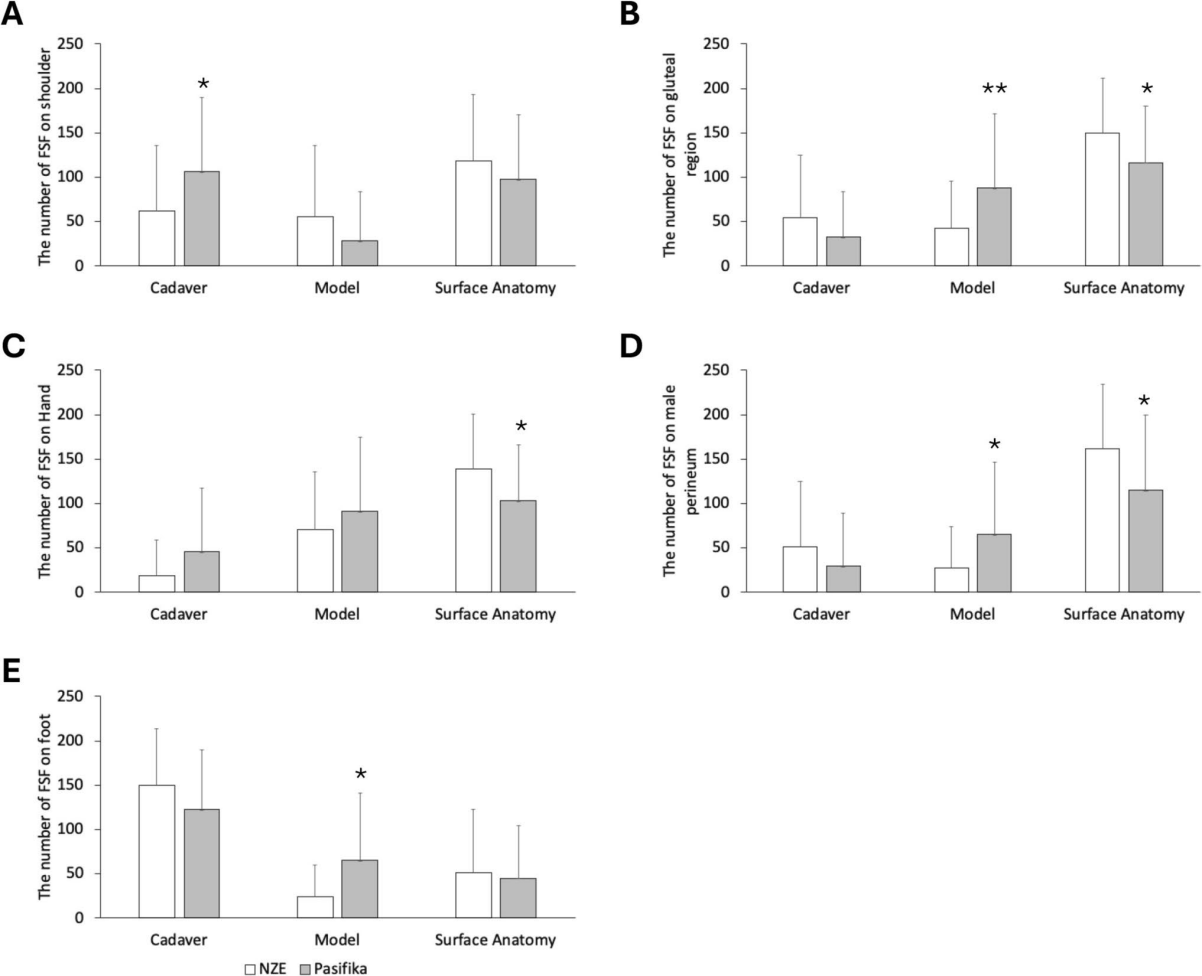
The total number of first–second fixations (FSF) on shoulder (**A**), gluteal region (**B**), hand (**C**), male perineum (**D**), and foot (**E**) images between NZE (white bars) and Pasifika (gray bars) students. Students viewed three images (cadaver, plastic model, surface anatomy) simultaneously for each region. Pasifika students had more FSF on the cadaver image of the shoulder (**A**) and more FSF on plastic model images of the gluteal region (**B**), male perineum (D), and foot (E) than NZE students. In addition, Pasifika students had fewer FSF on surface anatomy images of the gluteal region (**B**), hand (**C**), and male perineum (**D**) than NZE students. *Significantly different from NZE; *P* < 0.05; **P < 0.01

No significant interaction effect of ethnicity and image type on the total number of fixations in 15 s was found for all body regions. There were significant interaction effects of ethnicity and image type on students’ FSF to five regions: the hand (*F*(2,180) = 4.25, *P* = 0.016), gluteal region (*F*(2,180) = 6.80, *P* = 0.001), shoulder (*F*(2,180) = 4.36, *P* = 0.014), foot (*F*(2,180) = 4.76, *P* = 0.010), and male perineum (*F*(2,180) = 5.63, *P* = 0.004) (Fig. 1). Pasifika students fixated more on the deceased donor image of the shoulder region (*P* = 0.020), as well as the plastic model images for the gluteal region (*P* = 0.006), the male perineum (*P* = 0.040), and the foot (*P* = 0.011) compared to NZE students. Furthermore, Pasifika students had fewer fixations on the surface anatomy images of the gluteal region (*P* = 0.045), hand (*P* = 0.036), and the male perineum (*P* = 0.011) than NZE students. FSF to other regions were comparable between ethnicities.

**Fig. 2:**
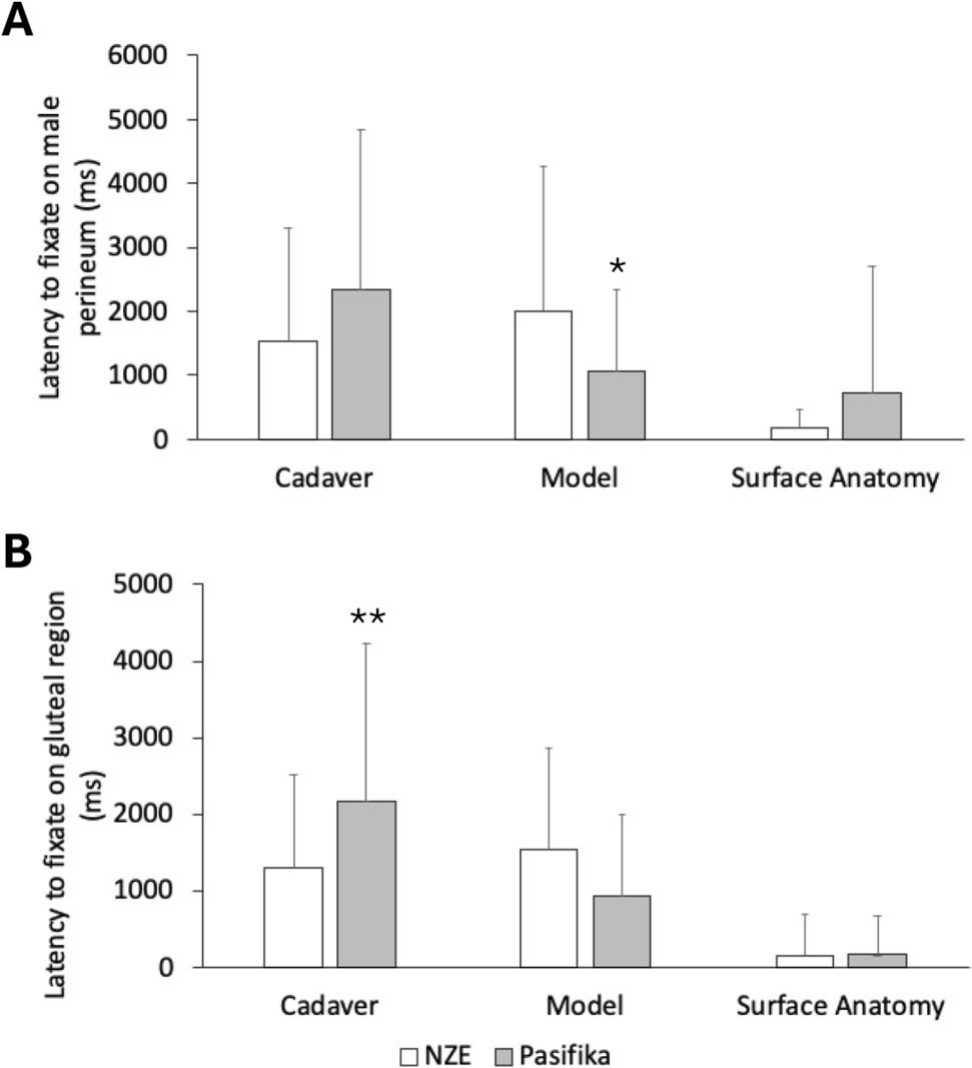
Comparison of latency (milliseconds) to fixate on the male perineum (**A**) and gluteal region (**B**) images between NZE (white bars) and Pasifika (gray bars) students. Students viewed three images (cadaver, plastic model, surface anatomy) simultaneously for each region. Pasifika students had a shorter latency to fixate on the plastic model image of the male perineum (**A**) and a longer latency to fixate on the cadaver image of the gluteal region (**B**). *Significantly different from NZE; *P* < 0.05; **P < 0.01

Significant interaction effects of ethnicity and image type were found on the latencies to fixate on the male perineum (*F*(2,180) = 4.10, *P* = 0.018) and the gluteal region (*F*(2,180) = 5.57, *P* = 0.004) (Fig. 2). The latency to fixate on the plastic model image of the male perineum (*P* = 0.044) was shorter, whereas the latency to fixate on the deceased donor image of the gluteal region (*P* = 0.006) was longer in Pasifika students than in NZE students. Latencies to fixate on other regions were comparable between ethnicities.

Our finding on regions which are considered *tapu* (gluteal and perineum) suggests that the comfort levels or cultural perceptions of the regions vary between the two ethnicities. As previously described in another publication [4], Pasifika students may not be comfortable in viewing nudity, given that many grow up in a religious environment, and may subconsciously avoid focusing on images from these regions.

The less attention to surface anatomy images by Pasifika students may also be related to the fact that all models were of European background. Past studies indicate that people tend to fixate on images of people of the same ethnicity or skin tone [11, 12]. Potentially, Pasifika students may engage more with surface anatomy images if the models had darker skin complexion that reflects their own ethnic background.

### Implications and Significance

Our finding reveals significant cultural variation that may influence how NZE and Pasifika anatomy students engage with anatomical images, underscoring the need for a culturally sensitive approach in educational settings. The integration of virtual reality or three-dimensional models for sensitive anatomical content may potentially mitigate the discomfort with traditional methods using deceased human donors. A future study could potentially compare students’ attention to real-life models or deceased donors, and how these data are associated with their academic performance. Moreover, our finding underscores the importance of cultural sensitivity training for educators, enabling them to facilitate discussions and teaching in a manner that respects and acknowledges cultural values concerning the human body.

### Limitations

Our study has several limitations. The sample size was relatively small and thus limits the statistical analyses that we could perform. Furthermore, participants were anatomy students; the results may not be generalizable to students who are not studying anatomy. Although all NZE students were of one ethnicity, we recruited Pasifika students with multiple ethnicities because the number of Pasifika students in our Department is few. This may add a confounding factor in our result.

## Conclusion

To our knowledge, we provide the first objective evidence on ethnic differences in visual attention to anatomical images. The findings underscore the need for universities to develop more effective anatomy teaching strategies for students who face challenges in learning anatomy.

## Data Availability

Data are not deposited in a repository.
